# Perceptual Broadening Leads to More Prosociality

**DOI:** 10.3389/fpsyg.2018.01821

**Published:** 2018-09-25

**Authors:** Sumitava Mukherjee, Narayanan Srinivasan, Neeraj Kumar, Jaison A. Manjaly

**Affiliations:** ^1^Department of Humanities and Social Sciences, Indian Institute of Technology Delhi, New Delhi, India; ^2^Centre for Cognitive Science, Indian Institute of Technology Gandhinagar, Chandkheda, India; ^3^Centre of Behavioural and Cognitive Sciences, University of Allahabad, Allahabad, India

**Keywords:** global local processing, scope of attention, prosocial, donation, decision making

## Abstract

A link between perceptual processing styles and (pro)social behavior has gathered supporting empirical evidence to show that people raised or trained in traditions of collectiveness, compassion, and prosocial beliefs are biased to the global level in perceptual processing. In this research, we studied the reciprocal link – whether contextually broadening perceptual scope of attention via global processing could make people more prosocial. We hypothesized that global processing linked previously to an interdependent compassionate self-orientation would make people more prosocial, compared to local processing. Four experiments manipulated perceptual scope through a Global-Local task using hierarchical stimuli. It was found that participants who performed a global processing perceptual task volunteered to donate more money across different donation frames, compared to those who performed a local processing task. While previous research showed prosocial mindsets lead to perceptual broadening, the current results suggest that perceptual broadening also leads to more prosociality, thus establishing a reciprocal link between perceptual broadening (attentional scope), and acting prosocially. It is proposed that perceptual scope of attention is one of the generic cognitive processes that underlie prosocial decisions. Explanations based on scope of attention can potentially be used as a framework that enables researchers to link the effects of different contextual cues on prosocial decisions.

## Introduction

People engage in prosocial behaviors/judgments relating to actions which are helpful to others. We are also encouraged by non-profit organizations and governments to help those in need by donating money or volunteering time using multiple contextual cues. They are often intended to promote compassion, empathy, and social connectedness. Given its importance in real life, prosocial decision making has become an active domain of interdisciplinary research spanning psychology, economics, neuroscience, sociology, philosophy, and political science (Oppenheimer and Olivola, 2010).

Many prosocial behaviors are complex decisions where explicit gains for the giver may not exist. Although multiple factors modulate donation decisions, fundamental cognitive processes, or mechanisms involved in such decisions have not been investigated extensively.A significant number of studies have looked at the effect of contextual factors like awe (Rudd et al., 2012), hunger(Briers et al., 2006), and happy moods (Anik et al., 2009) on prosocial appeals. However, we need to identify and understand domain-generic cognitive processes that could underlie a large number of contextual factors. In this research, we suggest and investigate one such domain-generic process – attentional scope linked with content-free perceptual styles of global-local processing (Friedman and Förster, 2010).

Prosocial decisions require attentional control mechanisms (Logan and Gordon, 2001) that are in-turn linked to, among other things, perceptual levels of global or local processing A series of studies have established a link between compassionate social connectedness and perceptual broadening. Those who undergo compassion meditation (Fredrickson et al., 2008; Leiberg et al., 2011; Weng et al., 2013) and Buddhist meditative techniques highlighting interdependent living (Colzato et al., 2010) and those who hold ideas of sovereignity (Colzato et al., 2008) show increased solidarity, compassion and social responsibility. They also show a larger global precedence bias compared to controls (Fredrickson et al., 2008; Colzato et al., 2010) possibly because such social practices broaden the mind’s horizon and accordingly induces a fundamentally broad attentional scope. Thus, these studies show that people exposed to beliefs about interdependent selves, solidarity, empathy, perspective taking, and compassion that are often key drivers of prosocial behavior (Leiberg et al., 2011; Decety et al., 2015) have a bias toward broad (or global) level of perceptual processing. Solidarity, empathy, and perspective taking could be positive emotions that expand both thoughts and actions of a person according to Broaden and Build Theory of positive emotions (Fredrickson, 2004). Such conceptual broadening might have led to or is closely coupled with perceptual broadening. Thus, attentional scope can be broadened at both the perceptual and conceptual levels of processing.

The link in the other direction – whether perceptual broadening can lead to prosociality has not been investigated in detail. Supporting literature has, however, suggested that a broad scope could integrate the self through shared action representations (Colzato et al., 2013) and create more interdependent relational thoughts (Nussinson et al., 2012). This in turn can potentially enhance perspective taking and feelings for others (Lamm et al., 2007). A broad scope (via global processing) has also been linked with thoughts about optimism, tranquility, social relationships, and empathy for others (Fredrickson et al., 2008).

From these findings, we suggest a new link: inducing a broad perceptual scope might lead to more prosocial behavior, compared to a narrow scope. We hypothesized this link as global processing yields a broader integration of the mind with others that can result in more empathy (Fredrickson et al., 2008; Colzato et al., 2013), which should lead to more prosocial behavior compared to local processing. Previous findings suggested a link in the exact opposite direction: higher prosocial mindsets lead to broader (global) perceptual bias. Taken together, these results would then mean there is a bi-directional link between perceptual broadening and prosociality, suggesting an interesting relation between perception, and prosocial decision making.

Four experiments were performed to investigate whether empirically manipulating perceptual scope can impact prosocial decisions. In all the experiments, perceptual scope was manipulated using global-local stimuli. A global processing style adopts a holistic, “zoomed-out,” broad scope of attention in comparison to a detailed, local “zoomed-in” perspective tuning people to the “whole” while local processing makes them attend to the “parts.” In the lab, the global-local task using hierarchical letters or shapes (Navon, 1977) has been extensively used for more than three decades to manipulate perceptual scope of attention. Such processing styles based on global versus local processing induce potentially content-free domain-generic processing strategies (procedural priming) that carry over to subsequent tasks, influencing both preference and memory (Srinivasan et al., 2013), critical for decision making. In a typical task, composite hierarchical stimuli are presented where a large letter or figure is made up of small letters but semantics is matched such that those in either the global or local groups see an equal number of letters. Of note here, these perceptual stimuli induce a processing style – “how” something is processed (procedural priming) and is not related to semantic content.

The first experiment was aimed to study and demonstrate the basic effect – would asking people to perform a global processing task that broadens attentional scope lead them to donate more money (compared to a group that does local processing)? Participants were presented with an appeal for donating money and then asked to perform a global-local task before responding to the appeal. In the second experiment, participants were asked to donate money after they performed the global-local task. Either the goal was introduced followed by manipulation of perceptual scope (Experiment 1) or vice-versa (Experiment 2). Experiment 3 tested the effect for a single victim using slightly modified appeals where one had an approach while the other had an avoidance orientation. In Experiment 4, we used an anonymous altruistic giving game based on the norms of fair resource distribution to test validity of the findings when the victim is not a poor person. These experiments were among the first to find the effect of perceptual broadening of attentional scope (using global-local processing) on prosocial donations.

## Experiment 1

### Participants

Ninety undergraduate students (*M*_age_ = 20 years, Females = 40, *SD* = 1.9 years) volunteered to participate in this study without any compensation. All were non-psychology engineering majors who took their courses in English. Participants were run individually in a closed room and all information was presented on a computer in English language.

### Stimuli and Procedure

**FIGURE 1:**
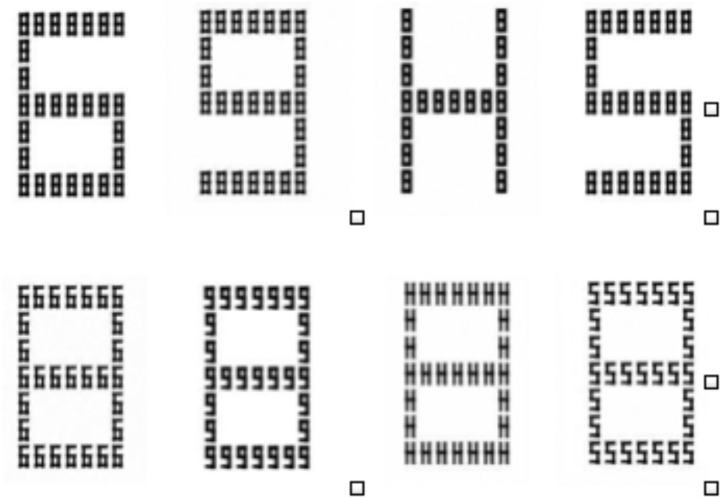
The Navon letters were all hierarchical in nature. The top row shows global (S, H, 6, and 9 at the global level with 8 at the local level) and bottom row shows local (S, H, 6, 9 was at the local level with 8 at the global level) stimuli.

The hierarchical stimuli used in the global task consisted of S, H, 6, and 9 at the global level with eight at the local level. Similarly, the stimuli used in the local task contained S, H, 6, and 9 at the local level with eight at the global level (see **Figure 1**). Global letters subtended 3.7°× 5.5 while local letters subtended 0.35°× 0.65. Stimuli were presented on a monitor and response was obtained using the keyboard.

Before the experiment, we showed them the global or local letters and explained that they have to count them – either global or local depending on the group a participant was allocated in. All participants were appraised about the counting task and we asked them to practice counting in a short session (12 trials). Participants were told that the experiment was on “human decision making.” They were instructed to read a social scenario that would appear on the screen, and then perform an unrelated “digit counting task” after which they could state their preferences related to the social scenario. Please note that they were not informed that a donation appeal would be presented. After the experiment was started, the following text introduced the social scenario with a donation appeal:

We would like to request you all to consider donating some money for the poor children living in nearby slums. You can help make their life better. We shall gift them what you give so that they could buy something for themselves. As part of this exercise, we need to know your preferences for this donation drive. Please think how much amount you would like to donate. However, while you think over, we require you to count some digits (as explained before). After you finish counting, state how much money you might be willing to donate for the poor children if asked for at a later date. Now, you need to fill up the amount only. We shall collect the money from you at a later date.

**FIGURE 2:**
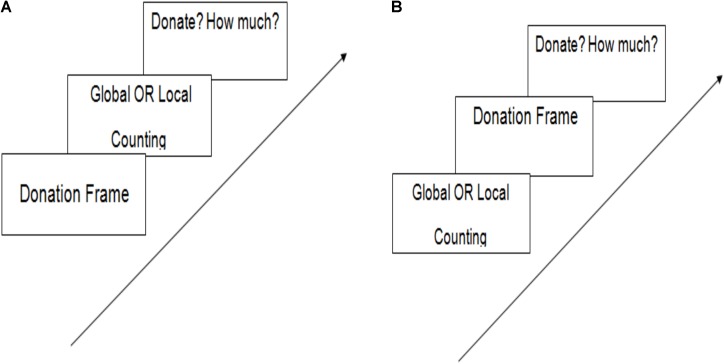
**(A)** Steps in Experiment 1 and 4. **(B)** Steps in Experiment 2 and 3.

After being asked to think about those people for 1 min, participants counted digits purported to be a distracter task but that task was actually used to manipulate attentional scope. The global group (*n* = 45) performed the task at the global level while the local group (*n* = 45) performed the task at the local level. The hierarchical letters were presented consecutively for 500 ms at the center of the screen followed by a blank screen for 500 ms. Total presentation time for the letters was 3 min (180 trials). In both the global and local tasks, participants were asked to count the number of times “S” appeared on the screen (with the correct answer ranging from 43 to 47 out of 180 occurrences). All participants had to count the letters at the appropriate level ignoring the other level (which does not change). After completing the counting task, they stated the count of the letters and were then asked to enter the amount (in Indian Rupees- INR) they were willing to donate for the poor children or 0 if they did not want to donate (see **Figure 2A**). They were also informed that it is a one-time donation and they need to put in the amount, which we shall collect at a later date. If they did not want to donate, they were asked to put 0 as donating was purely voluntary.

**FIGURE 3:**
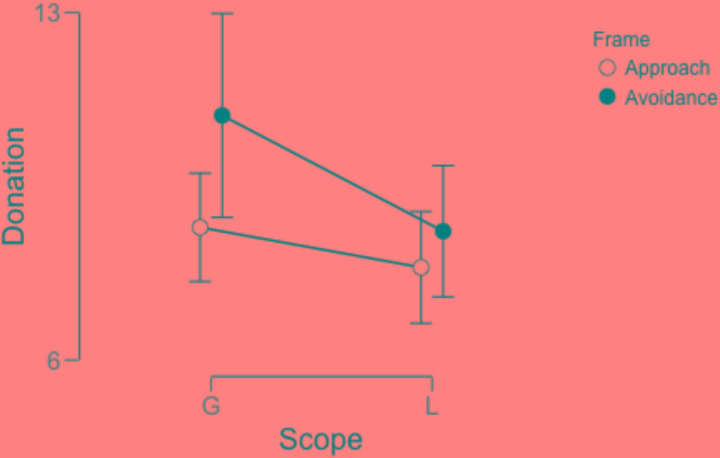
Donation amounts plotted for the different groups. The error bars show 95% CI.

### Results

The donation amounts were transformed using a square root function for analysis (Oppenheimer and Olivola, 2010; Huffmeijer et al., 2012)^1^. Mean square root of donations by global group was 9.06 (*SD* = 5.08) whereas for the local group it was 6.55 (*SD* = 3.74), Mean difference = 2.50, 95% CI [0.61–4.38], *F*(1,87) = 6.96, *p* = 0.01, η_p_^2^ = 0.07 including error in counting as a covariate^2^. These results agreed with our basic prediction that global processing would result in more donations compared to local processing. In Experiment 1, we manipulated the perceptual scope after the goal was set whereas the follow up experiment manipulated attentional scope before asking for donations to test whether the global-local effect on donations is replicable even when there is no previous goal set.

## Experiment 2

This experiment presented information about poor people without informing the participants that they shall be asked to take a decision to donate later. As donations have been argued to follow a two-step process in which the first is a binary decision about donation (to donate or not) and the second decision is a question about the amount of money to donate (Dickert et al., 2011), we incorporated it in our experiment. Participants also reported their emotions related to sympathy and mood management (Small et al., 2007; Dickert et al., 2011) to see whether perceptual broadening also affects overt reporting of emotions.

### Participants

One hundred and eight undergraduate student volunteers (*M*_age_ = 20.5 years, *SD* = 2.2, Females = 70) participated in the experiment.

### Stimuli and Procedure

The experiment was presented as a study on decision making to understand how information is processed. Perceptual scope of attention was manipulated between subjects where one group of participants saw global letters and another group saw local letters as in Experiment 1. At the beginning of the experiment, participants were presented with a short paragraph regarding the poor workers’ children in their college campus and asked to think about them for a minute. We did not mention that they would be asked to donate because we wanted to see whether processing styles influence fairly immediate donation decisions (**Figure 2B**).

The following text was presented:

“You can easily see that our campus has many poor workers living with their children. These children live in mud and cold without shoes, good clothing etc. which also make them sick. We need your help to make their lives better. We would require you think about these poor children and tell us actually how you feel and whether you would want to act towards improving their lives. Take some time (about 1 minute) to recollect your memory about these children’s lives. Press any key to continue after you have thought about them.”

After reading the passage above, one group was presented with only global letters (global group, *n* = 50) and another group saw only local letters (local group, *n* = 58). The global and local conditions used the same four composite global or local letters, respectively, as in Experiment 1.

After they entered the count of letters, we asked them whether they would want to donate any money for the poor workers’ children that they thought about (decision to donate: Y or N). Those who said “Y” for “yes” were asked to enter the amount that they would like to donate (donation amount). We also asked some affective questions (taken from Kogut and Ritov, 2005; Dickert et al., 2011) to probe whether the global-local processing influences self-reported affect. Sympathy was measured by asking (a) After reading about the poor workers’ children, how worried, upset and sad did you feel?, (b) How much sympathy and compassion did you feel toward these poor workers’ children?, and (c) How strongly do you feel overall for these poor workers’ children? One item measured self mood-management (Dickert et al., 2011) based on the hypothesis of warm-glow which suggests that people donate to feel good themselves: (d) How much better would you feel by donating money for these poor workers’ children? All responses were made on a 100-point (1 = not at all, 100 = very much) sliding scale with random initial positions of the mouse pointer. Later, the scores for questions a-c were aggregated for sympathy.

### Results

Donations were square root transformed. Three participants were excluded due to large errors in counting letters (>15%) and one participant was an outlier with respect to donation amount (>3 SD).

Out of 104 participants (global = 48, local = 56), 25 participants (5 in global and 20 in local) did not opt to donate money for the charity appeal and data from 2 participants were not recorded fully. More participants in the global group decided to donate compared to the local group (*n* = 77, global = 42, local = 35), a difference that was statistically significant (*p* = 0.0028, Fisher’s exact test). Among those who donated, the global processing group donated more (M_global_ = 12.72, *SD* = 5.30) compared to the local group (M = 8.63, *SD* = 3.53), Mean difference = 4.05 95% CI [1.91–6.19], resulting in a significant effect of perceptual scope *F*(1,74) = 14.18, *p* = 0.001, η_p_^2^ = 0.16, with counting errors as a covariate. There was no effect on questions about sympathy, *F*(1,74) = 0.56, *p* = 0.45 or on mood management, *F*(1,74) = 1.78, *p* = 0.18. These results showed that both when a goal is set (Experiment 1) or not (Experiment 2), performing a global processing task increased donations, in comparison to a local processing task. No effect of global-local processing was observed on affective ratings perhaps because manipulating attentional scope need not influence consciously reported affective states (Friedman and Förster, 2010) or because the affective emotions related to sympathy and mood management does not render itself easily to subtle manipulations of perceptual processing.

## Experiment 3

Donation appeals are framed for both groups and for single victims. Both of the previous experiments presented the appeal for a group. In donation appeals where an individual is presented along with a group of victims, local processing enhanced the preference for individual assistance in comparison to giving to the victims as a collective group (Oceja et al., 2017). However, it is not clear what would happen when the appeal for only a single victim is presented. This experiment tested whether the effect of global-local processing is generic, and hence even for single victims, global processing should result in more donations.

Further, framing of messages can interact with global versus local processing modes. Förster and Higgins (2005) manipulated processing styles and asked participants to assign price for a cup. After global processing, participants assigned a higher price for a mug when the appeal was framed in a promotion orientation (e.g., what would you gain by choosing it) while after local processing, they assigned a higher price when the appeal was framed in an avoidance orientation (e.g., what would you lose by not choosing). In a previous study on donation, it was found that when both promotion and prevention appeals were presented simultaneously and participants had to split an amount between both, global processing yielded more donations to the approach oriented framing compared to the avoidance oriented one (Mukherjee et al., 2014). However, in cases where a single appeal is presented in-line with previous experiments reported in this paper, we intended to test whether global processing result in more donations for both approach and avoidance oriented appeals or only for an approach oriented one. Thus, we presented appeals for a single victim in both an approach and an avoidance oriented framing to examine whether global processing would show the same effect or there are possible boundary conditions.

### Materials and Methods

#### Participants

One hundred twenty eight undergraduate student volunteers (*M*_age_ = 19.5 years, *SD* = 2.1 years, Females = 54) participated in the experiment.

#### Stimuli and Procedure

The procedure was exactly similar to Experiment 2 but used a 2 (processing style: global vs. local) × 2 (framing: approach versus avoidance) between-subject factorial design. The global and local conditions used the same four composite global or local letters as in previous experiments. First, participants were presented with a short appeal for a single poor worker’s girl in their college campus and asked to think about the person for a minute. They saw the following appeals [approach] or [avoidance] randomly allocated to each participant:

You can easily see that our campus has many poor workers living with their children. Mona is a child of a poor worker living in mud without shoes, good clothing etc. which also make her sick. You are required to think about this poor child Mona and tell us actually how you feel. [Approach: If you act, her life will become better] [Avoidance: If you do not act, her life will become worse.] Take some time to recollect your memory about such a child. Press any key to continue after you have thought about such a child.”

After reading each of the appeals, one group was presented with a global task based on letters that varied only at the global level and the other group was presented with a local task where letters varied only at the local level as before. They entered the count and whether they would want to donate any money (decision to donate: Y or N). Those who said “Y” for “yes” were asked to enter the amount that they would like to donate (donation amount), followed by the affective questions asked in Experiment 2.

### Results

Five participants were removed because of large errors in counting (>15%) and 8 participants were removed because their donation amounts were outliers (>3 SD). Donations were transformed using a square root function. Twenty-five participants (global = 14, local = 11, n.s., Fisher’s exact test) did not donate.

Ninety-seven participants opted to donate some amount of money. To account for the different frames, we conducted a 2 (global vs. local) × 2 (approach vs. avoidance frame) ANOVA with counting errors as a covariate. There was a main effect of processing (**Figure 3**) where the global group donated more than the local group, (M_global_ = 9.74, *SD* = 3.75; M_local_ = 8.28, *SD* = 2.88), Mean difference = 1.46, 95% CI [2.16–0.76], *F*(1,92)* =* 4.27, *p* = 0.04, η_p_^2^ = 0.04. We also observed a main effect of framing where the avoidance frame yielded more donations (M_approach_ = 8.24, SD = 2.74; M_avoidance_ = 9.78, *SD* = 3.88), Mean difference = -1.53, 95% CI [-2.23–0.83], *F*(1,92) = 4.81, *p* = 0.03, η_p_^2^ = 0.04. There was no significant interaction between processing style and framing of message. This could potentially be due to multiple reasons but one important difference between Mukherjee et al. (2014) and this study is the nature of the appeals. While there is only one appeal in this study, Mukherjee et al. (2014) had two competing appeals to which money had to be divided. We did not observe any effect of global-local processing on the affective questions about sympathy or on mood management (all *p* > 0.5).

The framing did not interact with levels of processing and similar effect was obtained with both types of framing. This showed that the direction of the effect was similar across different donation appeals even for a single victim with global processing linked to higher prosocial acts measured using monetary donations.

## Experiment 4

All the previous experiments used the poor as a victim whose relative socio-economic position was lower compared to our participants. To further test whether the effect of prosociality is more general, in this experiment, the appeal stressed that the victim is one among them such that the socio-economic backgrounds matched. We used a redistribution game task consisting of an unfair resource distribution scenario, which participants can change by donating their money. Specifically, the scenario involved an economic decision requiring costly redistribution of own funds to change a previous unfair transaction. The redistribution game used here was previously operationalized as a measure of compassion through altruistic distribution of one’s own resources (Weng et al., 2013). Compassion is one of the primary elements of altruistic behavior (Decety et al., 2015) defined as the emotional response of caring for others and wanting to help sufferers (Weng et al., 2013). The measure has also been validated by Weng et al. (2013) where the authors found that those who donate more in this game also endorse greater empathetic concerns in the test for prosocial traits measured using the empathetic concern scale. The task thus tested whether global processing results in a compassionate prosocial decision in an unfair situation, which is different from making a donation for a known condition of the world (like poverty). This would help generalize the previous effects from Experiments 1 to 3 more broadly for prosocial decision making.

### Participants

Fifty-eight young adults (*M*_age_ = 23 years, *SD* = 4, Females = 30) were recruited randomly by the experimenter from the campus. They belonged to both undergraduate and graduate programs and some were students from other institutes who were doing a summer internship.

### Stimuli and Procedure

**FIGURE 4:**
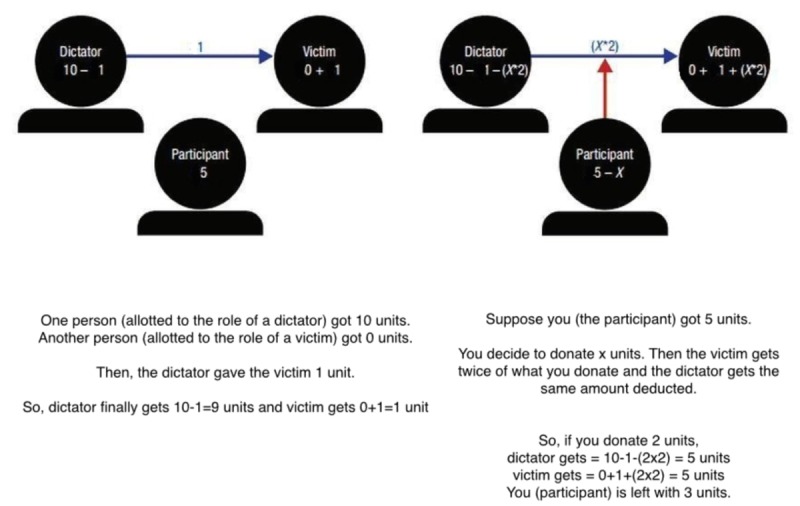
The compassion game presented to participants in Experiment 4.

Participants were first explained the global-local counting task and then the experimenter introduced them to an altruistic behavior game. Participants were explained the task in more detail by using example transfers different from what was to be used in the experiment (**Figure 4**) to reduce any deliberation before the actual experiment. The following information was then displayed on the screen:

“*Two people from this campus have been randomly selected and paired with each other. They were asked to play a game called a dictator game. In this game, one of the randomly chosen persons (the dictator) is given some money by the experimenter and is asked to split it between him/herself and the other person (the victim) according to one’s wish. The one who was allotted the role of a dictator was given 50 INR by the experimenters out of which the dictator decided to give 1 INR to the victim.*

You have a chance to change the previous transaction. We are giving you 20 INR. If you wish, you could donate a portion of that. Whatever money you donate, the dictator has to give twice that amount more to the victim.”

They were told that they have to think about what they would want to do. However, while they think over it, they needed to count some digits. One group performed a global counting task (*n* = 29) while the other group did a local counting task (*n* = 29). After finishing the counting task, they entered the count of letters and the amount that they wanted to donate out of 20 INR. Whatever money was left after donating was paid to the participants immediately after the experiment was over.

### Results

All participants had counted the digits reasonably correctly (errors < 15%) and hence no data was excluded from analysis. Square root transformed donations by global group (mean = 3.01, *SD* = 0.98) were more compared to the local group (mean = 2.40, *SD* = 1.24), showing a main effect of global-local processing, Mean difference = 0.59, 95% CI [0.30–0.88], *F*(1,55) = 4.08, *p* = 0.04, η_p_^2^ = 0.06 with counting errors as co-variate. This showed that the effect of global processing increasing monetary donations is not restricted only to those who are poor, but is a more general prosocial effect.

## General Discussion

Global processing increased monetary donations in a range of scenarios. When the appeal was presented first (Experiment 1), a goal was pre-decided while participants were exposed to the global or local stimuli. Later, we first asked participants to perform the global-local task and then showed the appeal (Experiment 2) and hence no explicit goal/task was set while perceptual processing was taking place. Further, we also modified the appeal from being directed at a group to being about a specific person in Experiment 3 with slight modifications in the motivational orientation (approach versus avoidance). We observed that across all three experiments, after global processing (linked perceptually to a broad scope of attention), donations were more compared to local processing (linked to a narrow scope). We also observed that these implicit processing styles are not overtly affective (Friedman and Förster, 2010) and did not influence affective self-reports about sympathy or mood management while at the same time influencing prosocial actions. A follow up experiment (Experiment 4) replicated the basic finding and showed that for unfair situations, global processing resulted in redistribution of own funds toward a prosocial empathetic concern. This, we hypothesized could be because global processing results in a compassionate interdependent self orientation that should make people more prosocial, compared to local processing (Fredrickson et al., 2008; Nussinson et al., 2012; Colzato et al., 2013).

Multiple contextual cues that influence prosocial behavior can be seen in light of the current findings. Here we highlight a set of previous studies on prosocial behavior where cues and psychological states that can be linked to broad or narrow attentional scope have been found to impact prosociality in-line with the current research. First, fundamental motivational states like hunger reduce monetary donations (Briers et al., 2006) by narrowing the perceptual systems to the self at present to address immediate needs (Schaller et al., 2007; Kenrick et al., 2010). Motives which localize attention to the self along with states like anxiety, negative affect (Gasper and Clore, 2002),and avoidance orientations (Förster and Higgins, 2005) narrow the scope of attention and hence should result in less prosocial behavior. Second, positive mood has been demonstrated to enhance donations (Isen, 1987) and is linked with broadening of attention (Fredrickson and Branigan, 2005) and processing of positive emotional information has been linked to broad scope of attention (Srinivasan and Hanif, 2010; Srinivasan and Gupta, 2011). On the same lines, primes like awe (defined as perceptual vastness and possibly associated with broad scope) increase pro-social behavior (Rudd et al., 2012). It is thus plausible that ageneric cognitive mechanism underlying prosocial behavior is scope of attention. Both previous and future studies can adopt an explanatory framework based on attentional scope to consolidate findings and also predict new results. Future studies on the effect of different contextual cues on prosociality could predict that if the contextual cue is known to have a broadening effect on attention (compared to another which narrows scope), then it would result in more prosocial behavior. While we have discussed only perceptual broadening in this paper, an explanatory framework based on attentional scope (which can be both perceptual and conceptual; Friedman and Förster, 2010) can potentially enable researchers to link the effects of contextual cues on prosocial decisions.

At an empirical level, one limitation of the study is that even though the global-local hierarchical stimuli have been used for decades since Navon (1977), we used the exact same stimuli to manipulate scope of attention in all the experiments. Our rationale to do so was to keep the manipulation constant and domain-generic (without semantic content; Friedman and Förster, 2010). Moreover, we always introduced the social scenario in all experiments before manipulating the scope of attention so that the thinking is oriented to the domain of social thought. This limits generalizing the effect of any other global-local percept on prosocial decision.

The second limitation is about possible moderators and mediators of the effect. While we have used random allocation of participants into experimental conditions, whether the effects of attentional scope would be similar across differences in culture (Masuda and Nisbett, 2001; Kühnen and Hannover, 2010), social distance of the victims (Waytz and Epley, 2012), socio-economic situations (Piff et al., 2010), personality traits (McClintock and Allison, 1989) and a host of other possible moderators and mediators including information about large groups versus individual victims (Dickert et al., 2012) is an open question. Future studies can chart out boundary conditions for the global-local effect reported here. The only other published work that looked into the relationship between perceptual scope and a related construct – empathy, reported local processing enhanced self-reported empathic concern (Woltin et al., 2011). One reason for deviation is perhaps related to the dependent variable. Woltin et al. (2011) measured empathy using a multi-item scale of concern where most of the items are worded in first person. We believe local processing should increase the focus on the self and hence one can rate self-related items in a questionnaire highly but it might not translate to actual behavior. We maintain that perceptual broadening is more closely associated to prosociality. A clear mechanistic explanation for the effect of global-local processing is not clear when applied to carry-over effects like these, but one direction to explain mechanisms need to look at functional manifestations of perceptual broadening. A broad scope of attention could integrate the self through shared action representations (Colzato et al., 2013) and create more interdependent relational thoughts (Nussinson et al., 2012).

The explanation linking perceptual broadening to broader prosocial actions fit with the literature that reported people trained in prosocial beliefs have a global bias (Colzato et al., 2010) and broadening the mind’s horizon induces a fundamentally broad attentional scope (Fredrickson et al., 2008). Further, meditative techniques that support open monitoring show a global attentional bias (Lippelt et al., 2014). Those who perform such practices show a greater widespread neuronal connectivity in the brain (Gard et al., 2015) linked to flexibility, mental health and well-being. Thus, it seems that the link from prosociality to perceptual broadening is also manifested structurally in the brain. A reverse link from perceptual broadening to prosociality as this study showed implies a bi-directional relationship between perceptual broadening and prosocial actions providing supportive complimentary evidence.

## Ethics Statement

The study was approved by the research review board and the doctoral advisory committee of SM at Indian Institute of Technology Gandhinagar. All participants provided written consent before voluntarily taking part in the study. The data was collected at Indian Institute of Technology Gandhinagar, Gujarat.

## Author Contributions

SM, NS, JM, and NK conceived the studies. NK and SM designed the experiments. SM and NK collected the data. SM and NK analyzed the data. SM and NS wrote the manuscript.

## Conflict of Interest Statement

The authors declare that the research was conducted in the absence of any commercial or financial relationships that could be construed as a potential conflict of interest.

## Footnotes

1 In all four experiments reported in this paper, the results do not change after converting the donations using a square root function.

2 The results do not change whether counting error is included or not for analysis. However, to be more conservative, we have used errors as a covariate in all analysis.
